# Metabolic Adaptations in Rapeseed: Hemin-Induced Resilience to NaCl Stress by Enhancing Growth, Photosynthesis, and Cellular Defense Ability

**DOI:** 10.3390/metabo14010057

**Published:** 2024-01-15

**Authors:** Xutong Lu, Dianfeng Zheng, Naijie Feng, Guangsheng Zhou, Aaqil Khan, Huimin Zhao, Peng Deng, Hang Zhou, Feng Lin, Ziming Chen

**Affiliations:** 1 College of Coastal Agriculture Sciences, Guangdong Ocean University, Zhanjiang 524088, China; 2 College of Plant Science and Technology, Huazhong Agricultural University, Wuhan 430070, China; zhougs@mail.hzau.edu.cn

**Keywords:** rapeseed, NaCl stress, hemin, physiology

## Abstract

This study aimed to investigate whether presoaking with hemin (5 μmol·L^−1^) could alleviate NaCl stress during rapeseed seedlings’ growth and its role in the regulation of photosynthesis. In this experiment, ‘HUAYOUZA 62 (HYZ 62)’ and ‘HUAYOUZA 158R (158R)’ were used as materials for pot experiments to study the morphology, photosynthetic characteristics, antioxidant activity, and osmoregulatory factors of seedlings under different salt concentrations, as well as the regulatory effects of hemin-presoaked seeds. Our findings revealed that, compared the control, NaCl stress inhibited the growth of two rapeseed varieties, decreased the seedling emergence rate, and increased the content of malondialdehyde (MDA), the electrolyte leakage rate (EL) and antioxidant enzyme activity. Hemin soaking alleviated the adverse effects of salt stress and increased plant height, root elongation and dry matter accumulation. Compared with all NaCl treatments, hemin significantly enhanced photosynthetic indexes, including a percent increase of 12.99–24.36% and 5.39–16.52% in net photosynthetic rate (Pn), 17.86–48.08% and 8.6–23.44% in stomatal conductivity (Gs), and 15.42–37.94% and 11.09–19.08% in transpiration rate (Tr) for HYZ62 and 158R, respectively. Moreover, hemin soaking also increased antioxidant enzyme activities, including superoxide dismutase (SOD), catalase (CAT), peroxidase (POD), and ascorbate peroxidase (APX), reducing the malondialdehyde, and thus resulting in the alleviation of oxidative damage caused by NaCl stress. Furthermore, hemin stimulated the formation of soluble protein, which effectively regulated the osmo-protective qualities. The current findings strongly elucidate that hemin soaking could effectively alleviate the negative impacts of NaCl stress by regulating the morphological, photosynthetic, and antioxidant traits. This study provides a new idea regarding the effect of Hemin on the salt tolerance of rapeseed, and provides a basis for the practical application of Hemin in saline–alkali soil to improve the salt tolerance of cultivated rapeseed.

## 1. Introduction

Globally, soil salinity is a severe environmental problem that harms agricultural production [1]. Currently, about 20% of the world’s arable land and half of irrigated croplands are exposed to soil salinity [2]. The salt-affected area in China is around 9.2 × 107 hm^2^, equivalent to 5% of the total land area [3]. The study has shown that a high salt concentration in soil can inhibit the normal growth and development of crops [4]. Salt can affect various physicochemical processes [5], such as osmotic stress, ion toxicity, ROS accumulation [6], and membrane lipid peroxidation [7], and ultimately reduce plant yield. In addition, salinity stresses markedly inhibited both ecological balance and crop productivity. The increasing demand for food is inhibited by the limited arable land and unstable environmental situations [8].

Rapeseed (*Brassica napus* L.) is an essential oil seed crop worldwide, and is a meal protein for humans and animal feed [9]. However, salinity stress affects rapeseed yield by retarding the seed germination, seedling growth and development, and grain quality of rapeseed [10,11]. Salinity inhibits the growth and development of rapeseed seedlings by affecting physiological traits such as photosynthesis and nutrient absorption through osmotic stress and disrupting ion homeostasis, resulting in a low grain yield [12]. Salt stress also leads to oxidative stress in oilseed rape, causing increased levels of reactive oxygen species (ROS) and inducing malondialdehyde production, which impacts cellular metabolism and causes physiological disorders in cell structure [13]. In oxidative stress, plants increase their resilience through antioxidant systems such as SOD, which catalyzes the decomposition of O^2−^ into O_2_ and H_2_O_2_, and CAT and POD reduce oxidative stress [14]. In addition, plants increase organic osmoregulatory substances such as soluble proteins through osmoregulatory mechanisms to maintain the stability of the intracellular environment, thereby protecting cells. Soluble proteins protect plant cell homeostasis under salt stress by balancing the cell solute and vacuole’s osmotic pressure with the external environment [15]. To a certain extent, applying plant growth regulators might overcome adverse environmental factors, improve crop stress resistance, enhance photosynthetic efficiency, and achieve a high level of grain production with better quality. For example, previous studies showed that prohexadione calcium enhanced the net photosynthetic rate of rice under salt stress and increased the activity of antioxidant enzymes, thus promoting plant growth [16].

Plant growth regulators are used in various ways to control plant growth and development. Hemin is the purified form of natural Heme in vitro, which is generally isolated and purified from animal blood. Hemin is a simple metal porphyrin compound formed by a complex comprising ferrous and protoporphyrin molecules [17]. It has been reported that Hemin acts as an inducer of heme oxygenase-1 (HO-1) at the transcriptional, translational and post-transcriptional levels [18], and can be used as a highly effective inducer to alleviate the damage caused by abiotic stresses (heavy metals, salinity, drought, etc.) [19]. Hemin treatment significantly alleviates lipid peroxidation in wheat cells, increases glutathione peroxidase activity, and significantly improves the salt tolerance of wheat seedlings [20]. Hemin improved the SOD, POD, and APX activities of tobacco seedlings subjected to salt stress. Hemin treatment can effectively enhance the activity of antioxidant enzymes and induce ion regulation to reduce ion accumulation and alleviate the damage that salt stress causes to tobacco seedlings [21]. The plant root system is the first organ to sense cadmium stress signals, and studies suggest that hemin can promote the formation of lateral roots of maize [22] and pak choi [23]. However, the mechanism hemin uses to alleviate NaCl stress in rapeseed remains unclear.

In summary, this experiment was conducted to study (1) the effects of NaCl stress on the growth of rapeseed seedlings and (2) the effects of hemin soaking on the growth of rapeseed seedlings under salt stress. From the perspective of exogenous regulation, the mechanism of salt-tolerant rapeseed varieties under NaCl stress and the regulation effect of hemin were analyzed using a growth regulator. This is expected to further reveal the salt-tolerance mechanism of oilseed rape, provide new ideas for the establishment of an oilseed rap, salt-tolerance, chemical-control cultivation project, and solve the technical problems in improving the salt tolerance of oilseed rape, which are difficult to implement using traditional agronomic measures.

## 2. Materials and Methods

### 2.1. Test Materials and Salinity Treatment

The current study used rapeseed varieties, i.e., HYZ62 (salt-tolerant) and 158R (medium salt tolerance) as the plant materials. The Huazhong Agricultural University, Wuhan, China, provided both materials. The test regulator was hemin, which Shanghai Changdeduo Agricultural Technology Co., Ltd. provided. The soil was a mixture of quartz sand:vermiculite:earthworm soil = 1:1:1.

### 2.2. Experimental Design

Pot experiment was conducted in 2022 in a solar greenhouse at Guangdong Ocean University. Uniform, full, uniformly sized rapeseed was selected, and the surface was sterilised with 3% hydrogen peroxide for 8 min and then rinsed 4–5 times with distilled water. After drying, seeds of both varieties were imbibed in (1) deionised water (control); (2) hemin (5 μmol·L^−1^) solution for 8 h (darkness, 20 ± 2 °C), with a seed-weight-to-solution-volume ratio of (*w/v*) 1:3, and the seeds were dried after 8 h via water absorption.

Seeds were sown in seedling trays (top opening 6.5 cm; bottom opening 3.55 cm; height 6.5 cm). Each seedling tray had 21 holes, with each containing about 250 g of test soil. The soil was treated with salt before sowing. Each hole was filled with 70 mL of salt solution (measured with a salinity meter, SMART SENSOR AR8212) and the salt concentrations were set at 0.3% (51.3 mM), 0.6% (102.6 mM), 1.2% (205.2 mM), and a blank control (water). Three seeds were sown in each pot and seedlings were thinned out when they reached 3–5 cm in height to ensure there were two seedlings per pot. Under normal lighting conditions, the treatment and control were repeated 3 times each, and the following 8 treatments were set up: 1. control (deionized water soaking); 2. hemin (5 μmol·L^−1^ hemin-impregnated seed); 3. 0.3%S (deionized water soaking + 0.3% NaCl); 4. hemin + 0.3%S (5 μmol·L^−1^ hemin-impregnated seed + 0.3% NaCl); 5. 0.6%S (deionized water soaking + 0.6% NaCl); 6. hemin + 0.3%S (5 μmol·L^−1^ hemin-impregnated seed + 0.6% NaCl); 7. 1.2%S (deionized water soaking + 1.2% NaCl); 8. hemin + 1.2%S (5 μmol·L^−1^ hemin-impregnated seed + 1.2% NaCl).

Samples were taken after 14 days of growth on days 0, 3, and 6 for the determination of physiological and biochemical parameters. Each sampling time was controlled from 7:30 a.m. to 10:30 a.m. to avoid the influence of environmental factors on the test results. The harvested oilseed rape seedlings were used to study different parameters, like growth index determination, photosynthesis and photosynthetic pigment determination, membrane stability, and antioxidants.

### 2.3. Measurement Items and Methods

#### 2.3.1. Germination Characteristics

Seedling emergence date: The number of days from sowing date to seedling emergence date was observed for each pot of seed, and the date of emergence was considered to be when more than half of the seedlings emerged from each treatment.

Seedling emergence: Seedling emergence was calculated for each treatment 7 days after sowing. Seedling emergence (%) = (number of seedlings/number of seeds) × 100.

Leaf moisture content = (leaf fresh weight - leaf dry weight)/leaf fresh weight × 100%.

#### 2.3.2. Growth Index Measurement

Morphological indicators of plants: (1) plant height and root length: measured by straightedge; (2) above-ground fresh weight and above-ground dry weight: weighed by universal balance; (3) leaf area: measured by Leaf Area Meter.

Biomass: Seedlings were cut from the stem base into above-ground and below-ground parts and weighed separately to determine fresh weight.

Dry matter: Seedlings are placed in an oven at 105 °C for 30 min and then dried at 80 °C to a constant weight. The dry weights of the above-ground parts and roots are measured using a universal balance and the water content of the leaves is calculated.

#### 2.3.3. Determination of Photosynthetic Pigments in Leaves

Determination of photosynthetic pigment content in leaves: according to the method of [24], 0.1 g of leaves from each treatment were immersed in 10 mL of anhydrous ethanol, left at room temperature, and protected from light for 24 h. The photosynthetic pigment content was determined by colorimetric method; absorbance values at 665 nm, 649 nm and 470 nm were determined. The pigment concentration (mg·L^−1^) was calculated according to the following formula:

Chlorophyll a (Chl a) = 13.95D665 − 6.88D649

Chlorophyll b (Chl b) = 24.96D649 − 7.32D665

Total chlorophyll content = Chl a + Chl b

Carotenoid (Car) = (1000 D 470 − 2.05 Chl a − 111.48 Chl b)/245

#### 2.3.4. Determination of Photosynthetic Characteristics of Leaves

Net photosynthetic rate (Pn), transpiration rate (Tr), stomatal conductance (Gs), and intercellular carbon dioxide concentration (Ci) of leaves were measured using a Li-6400 portable photosynthesis meter (LI-COR, Inc., Lincoln, NE, USA). Relative humidity was maintained at 70% during the measurements.

#### 2.3.5. Determination of Lipid Peroxidation and Membrane Permeability

According to the method described by [25], malondialdehyde (MDA) content was determined using the thiobarbituric acid method. A total of 0.5 g of fresh leaves was ground and then homogenised in 5% trichloroacetic acid (TCA) and 0.65% thiobarbituric acid (TBA). The mixture was boiled at 100 °C for 1 h and kept at room temperature to cool. After filtration, the absorbance of the supernatant was measured at 450 nm, 532 nm and 600 nm.

Determination of membrane damage index: determination of electrolyte leakage according to the method described by [26]. Weigh 0.1 g fresh leaves (functional leaf) and soak them in 10 mL deionized water at room temperature for 12 h. The conductivity (R1) is measured with a conductivity meter. The measured sample is then boiled in boiling water for 30 min, and the conductivity (R2) is measured after cooling. The relative vane conductivity (EL) is calculated using the following formula: EL = R1/R2 × 100%.

#### 2.3.6. Determination of Antioxidant Activity

To determine the enzyme activity, 0.5 g of fresh leaves was taken and ground in 10 mL of 50 mM phosphate buffer solution (PBS) (pH 7.8) for enzyme purification to obtain crude enzyme extract, and then the supernatant was used for the measurement of superoxide dismutase (SOD), catalase (CAT), peroxidase (POD), and ascorbic acid peroxidase (APX).

The activity of superoxide dismutase (SOD) was determined by the nitrogen blue tetrazole (NBT) color-developing method [27]. A total of 100 μL of the crude enzyme extract was added to 2.9 mL of enzyme reaction solution. The reaction mixture was placed under fluorescent light for 5 min and then the reaction was stopped in the dark. Subsequently, the absorbance was measured at 560 nm.

The guaiacol method detemined peroxidase (POD) [28]. The absorbance value of the reaction mixture at 470 nm was determined by the guaiacol method as described and counted every 30 s.

Ascorbate peroxidase (APX) and catalase (CAT) were determined by spectrophotometer [29]. CAT activity was calculated by measuring the decomposition of H_2_O_2_ at 240 nm and counted every 30 s. As the ASA was oxidised, APX activity was measured at 290 nm and counted every 30 s.

The soluble protein content was determined by the Coomassie brilliant blue G-250 method [30]. Absorbance values at 595 nm were determined by binding the proteins to the Caumas Brilliant Blue.

### 2.4. Statistical Analysis

Excel 2019 software was used for data processing, SPSS 19.0 statistical software was used for variance analysis, ANOVA was used to compare the three processing mean values, and Duncan’s multiple test was used for multiple comparisons. Origin 2021 software was used to draw graphs and make correlation analysis graphs.

## 3. Results

### 3.1. Effects of Hemin Soaking under NaCl Stress on Seedling Emergence Rate and Leaf Water Content

When the NaCl level increased, the days to emergence of both varieties increased (Supplementary Material Table S1). The results showed that the emergence rate was inversely proportional to NaCl levels and the lowest value was observed at 1.2% NaCl. Hemin-soaking treatment showed a consistently significant effect on the emergence rate.

**Figure 1:**
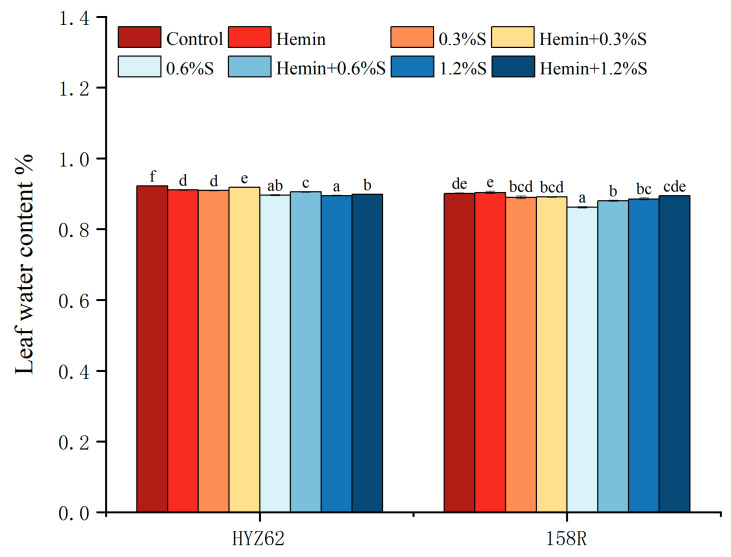
Effects of hemin on water content of HYZ62 and 158R leaves under NaCl Stress (14d). Mean ± SE of three replicates. Different letters indicate significant differences (*p* < 0.05).

Under NaCl stress, the water content of rape leaves decreased significantly (Figure 1). The water content of HYZ62 leaves was inversely proportional to NaCl concentration. The leaf water content of 158R first decreased and then increased with the increase in NaCl concentration, but was lower than that of control plants.

Similarly, hemin + 0.3%S, hemin + 0.6%S and hemin + 1.2%S treatments increased leaf water content by 1.0% and 0.15%, 1.0% and 2.12%, and 0.45% and 1.06%, respectively, in HYZ62 and 158R compared to all NaCl treatments. However, hemin treatment significantly reduced the leaf water content of HYZ62 compared to the control treatment.

### 3.2. Effects of Hemin Seed Soaking under NaCl Stress on Growth Performance and Biomass of Rape Plants

**Figure 2:**
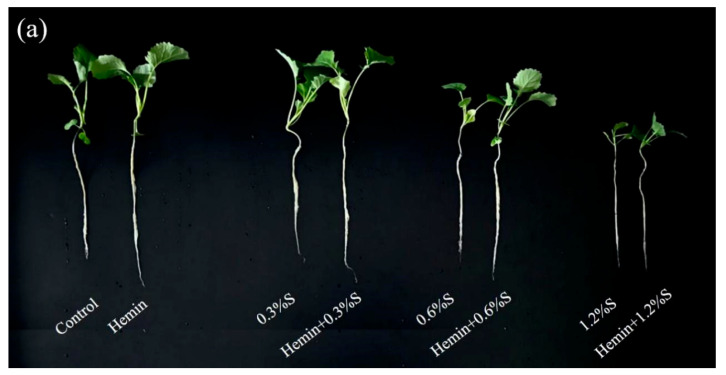

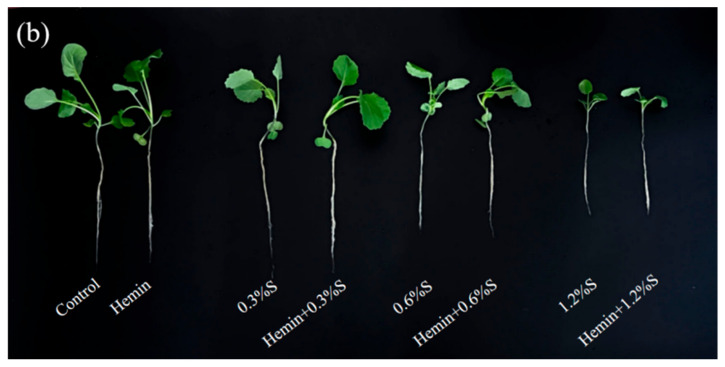
Effect of hemin on rape growth under NaCl stress (14d). Seedling morphology of HYZ62 (**a**) and 158R (**b**) after 14 days under salt stress.

NaCl stress had a significant effect on the growth characteristics of plant seedlings (Table 1, Figure 2), especially at 1.2%S, which was mainly characterised by reduced plant height, shortened root length and shrivelled leaves. The shoot and root lengths of the two oilseed rape varieties were gradually reduced with increasing NaCl concentration. The shoot lengths of HYZ62 were reduced by 35.45%, 15.40%, and 39.54%, and the root lengths were reduced by 20.63%, 18.29%, and 14.96%, on the 14th, 17th, and 20th days, respectively, of the stress at 1.2% S. The root lengths of the two oilseed rape varieties were reduced by 20.63%, 18.29%, and 14.96% on the 14th, 17th, and 20th days of the stress, respectively. Under 1.2% S stress, 158R showed 44.34%, 32.18% and 18.75% reduction in shoot length and 14.42%, 10.09%, and 11.27% reductions in root length at days 14, 17, and 20, respectively. The leaf area of both oilseed rape varieties was significantly reduced under NaCl stress (Table 1). The leaf area of HYZ62 decreased by 20.47 and 27.7% on days 14 and 17, respectively, but increased by 8.22% on day 20 with the 0.3%S treatment compared to the control. With 0.6%S and 1.2%S treatments, the leaf area of HYZ62 decreased by 14.93–52.03% and 31.71–62.12%, respectively. The leaf area of 158R increased with 0.3%S treatment but decreased in the other two concentrations of salt stress. However, the unfavourable effects of salt were significantly alleviated by stonemethanol soaking (Table 1, Figure 2). Compared to NaCl treatment, oilseed rape seedlings of both varieties showed a growth trend of higher plant height and longer root length after hemin soaking. In particular, both the shoot and root lengths of HYZ62 and 158R increased significantly with hemin + 1.2%S treatment. On days 14, 17, and 20, the shoot length of HYZ62 increased by 7.8%, 12.05%, and 14.5%, respectively, and the root length increased by 17.33%, 11.72%, and 13.35%, respectively, especially with the hemin + 1.2%S treatment. 158R increased by 22.58%, 12.9%, and 12.9%, respectively, and the root length increased by 22.58%, 12.9%, and 12.9%, respectively, in hemin + 1.2%S. The shoot length of 158R increased by 22.58%, 12.9%, and 20.71%, and the root length by 20.22%, 15.38%, and 28.43%, respectively. Compared with all NaCl treatments, the leaf area of HYZ62 seedlings tended to decrease, while that of 158R seedlings tended to increase.

**Figure 3:**
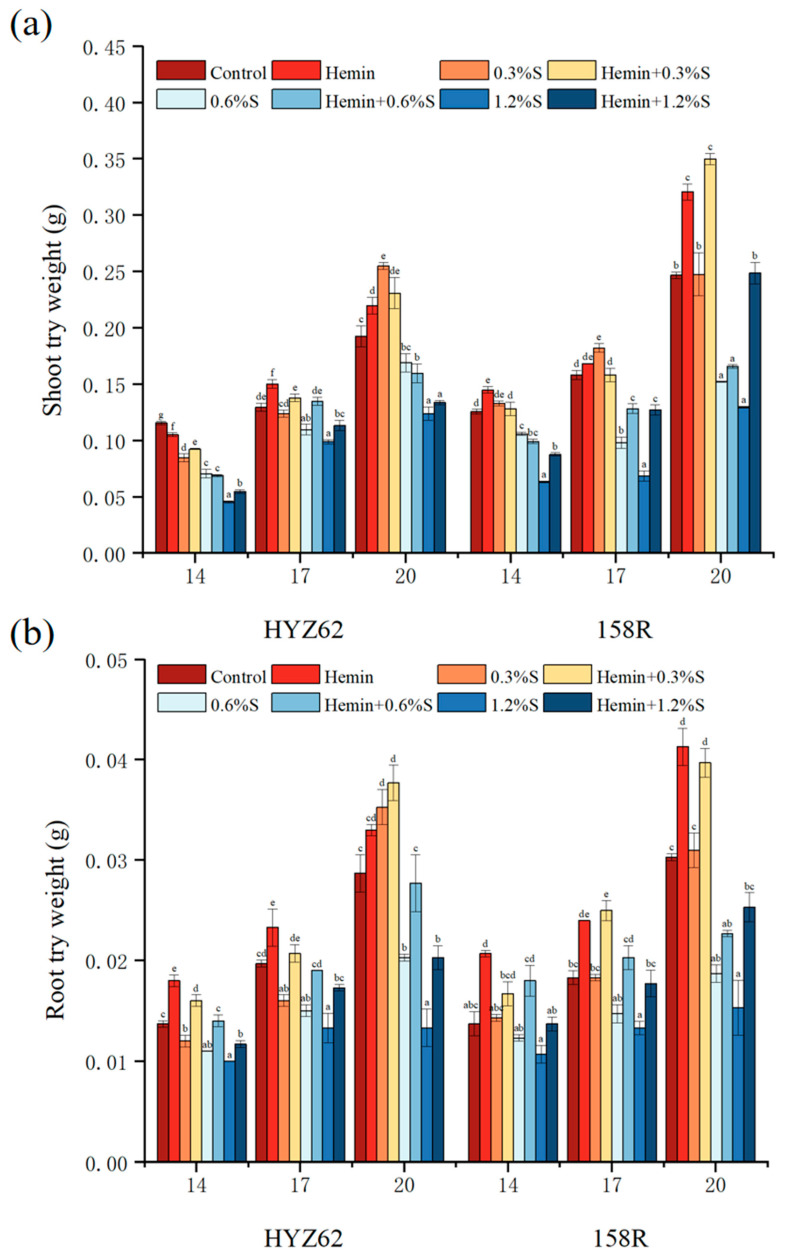
Effects of hemin on shoot dry weight (**a**) and root try weight (**b**) of HYZ62 and 158R rape seedlings under NaCl stress. Mean ± SE of three replicates. Different letters indicate significant differences (*p* < 0.05).

Compared to control, NaCl stress significantly reduced the biomass of the two rape varieties (Figure 3). In HYZ62, the shoot dry weight of 0.3%S treatment increased by 26.79% and 4.39% on the 14th and 17th days, but by 32.33% on the 20th day. The shoot dry weight of 158R with the 0.3%S treatment increased by 0.41~15.16%, indicating that low levels of salt stress can promote the plant dry matter accumulation. However, the shoot dry weight of both varieties significantly decreased under high-salt-concentration stress. Similarly, compared to the control, the shoot fresh weight of plants increased with the 0.3%S treatment but significantly decreased with the 0.6%S treatment and 1.2%S treatment (Table S2). After the hemin soaking shoot, the dry weight of HYZ62 and 158R decreased with 0.3%S treatment and 0.6%S treatment, which may be related to the decrease of leaf area. However, with the 1.2%S treatment, the shoot dry weight increased significantly after hemin soaking. The root fresh weight and root dry weight of the two varieties of rapeseed seedlings decreased under salt stress, but increased after hemin soaking. Compared to all salt treatments, HYZ62 increased by 6.8–33.33% with hemin + 0.3%S treatment, 26.67–36.45% with hemin + 0.6%S treatment, and 17–52.63% with hemin + 1.2%S treatment. 158R increased by 16.78~36.61% with hemin + 0.3%S treatment, 21.39~46.34% with hemin + 0.6%S treatment, and 28.04~65.36% with hemin + 1.2%S treatment.

### 3.3. Effects of Hemin Soaking on Photosynthetic Pigment Content of Rape Seedlings under NaCl Stress

**Figure 4:**
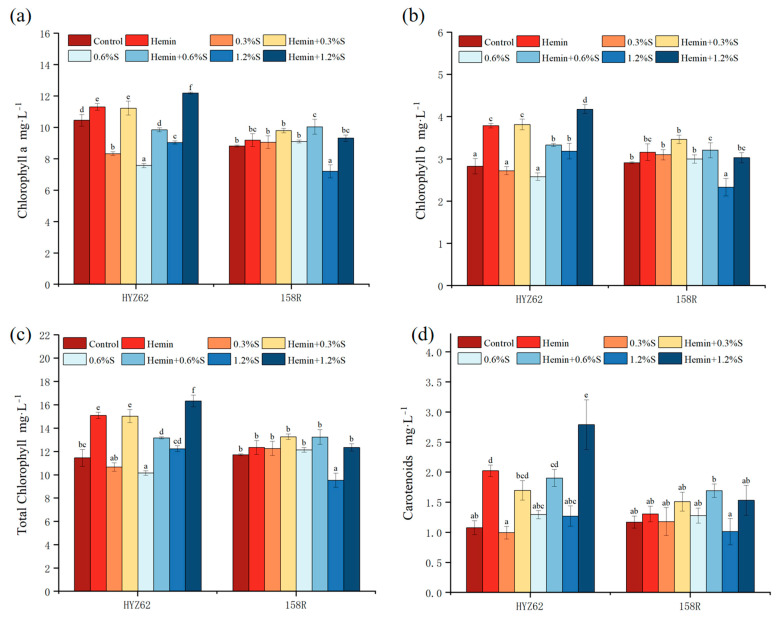
Effects of hemin soaking on chlorophyll a (**a**), chlorophyll b (**b**), total chlorophyll, (**c**) and carotenoid (**d**) contents of HYZ62 and 158R under NaCl stress (14d). Mean ± SE of three replicates. Different letters indicate significant differences (*p* < 0.05).

In HYZ62 varieties, salt stress resulted in a significant decrease in chlorophyll a content (Figure 4a). Compared to control, chlorophyll b slightly decreased with 0.3%S treatment and 0.6%S treatment but did not reach a significant level, while it significantly increased with 1.2%S treatment. The changes in total chlorophyll content and chlorophyll b content in HYZ62 under salt stress were consistent (Figure 4b,c). In the high salt concentration treatment, the carotenoid content of HYZ62 increased but did not reach a significant level (Figure 4d). However, the variation trend of 158R under NaCl stress is completely different from that of HYZ62. Compared to the control, chlorophyll a, chlorophyll b, and carotenoid in 158R increased with 0.3%S treatment and 0.6%S treatment but did not reach a significant level. In the 1.2%S treatment, chlorophyll a and chlorophyll b significantly decreased, and carotenoid content decreased but did not reach a significant level. In this study, the photosynthetic pigment content of hemin-soaking rapeseed seedlings was higher than that of water-soaked seedlings, with or without NaCl stress (Figure 4). In terms of total chlorophyll, hemin + 0.3%S, hemin + 0.6%S and hemin + 1.2%S of HYZ62 increased by 40.91%, 29.69%, and 33.44%, respectively, and 158R increased by 8.24%, 9.06% and 29.38% (Figure 4c). In terms of carotenoids, HYZ62 increased by 70.55% with hemin + 0.3%s, 47.05% with hemin + 0.6%s, and 83.18% with hemin + 1.2%s treatment, while 158R increased by 28.04%, 32.36%, and 51.2% (Figure 4d). The increase in HYZ62 photosynthetic pigment after hemin soaking was greater than 158R, which indicated that the regulation effect of hemin on HYZ62 photosynthetic pigment after hemin soaking was better than 158R.

### 3.4. Effects of Hemin Soaking on Photosynthetic Capacity of Rape Seedlings under NaCl Stress

**Figure 5:**
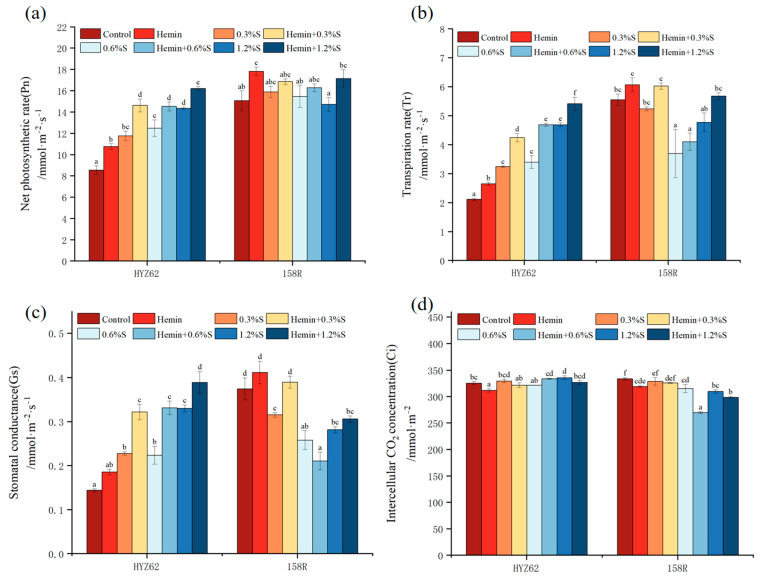
Effects of hemin soaking on net photosynthetic rate (Pn) (**a**), transpiration rate (Tr) (**b**), stomatal conductance (Gs), (**c**) and intercellular CO_2_ concentration (Ci) (**d**) of HYZ62 and 158R under NaCl stress. (14d). Mean ± SE of three replicates. Different letters indicate significant differences (*p* < 0.05).

Under NaCl stress, Pn, Tr and Gs of HYZ62 were significantly increased compared to the control; Ci was only significantly increased with 1.2%S treatment and slightly decreased with the other two salt treatments (Figure 5). The Pn of 158R increased with 0.3%S treatment and 0.6%S treatment, and slightly decreased with 1.2%S treatment, but did not reach a significant level compared to the control (Figure 5a). The Tr of 158R slightly decreased with 0.3%S treatment, significantly decreased with 0.6%S treatment and 1.2%S treatment, and decreased the most with 0.6%S treatment, decreasing by 33.43% (Figure 5b). The specific performance of Gs in 158R was that all NaCl stress significantly decreased compared to the control, and 0.3%S treatment, 06%S treatment, and 1.2%S treatment led to decreases by 15.75%, 31.06%, and 24.74%, respectively (Figure 5c). In addition, Ci decreased by 1.5% with 0.3%S of 158R, 5.59% with 06%s, and 7.19% with 1.2%S compared to control (Figure 5d).

Compared to the control, Pn, Tr, and Gs of HYZ62 and 158R slightly increased with hemin treatment, while Ci significantly decreased (Figure 5). Compared to NaCl treatment, the Pn of HYZ62 and 158R was significantly enhanced after hemin soaking. The hemin + 0.3%S treatment, hemin + 0.6%S treatment, and hemin + 1.2%S treatment of HYZ62 led to increases of 24.36%, 16.27%, and 13.0%, and treatment of 158R led to increases of 6.08%, 5.39%, and 16.52% compared to water-soaking treatment (Figure 5a). In Tr, HYZ62 increased by 30.83%, 37.94%, and 17.86% with 0.3%S, 0.6%S, and 1.2%S treatments after hemin-preimpregnated seed, and 158R increased by 14.95%, 11.09%, and 19.08%, respectively. Hemin + 0.3%S, hemin + 0.6%S, and hemin + 1.2%S treatments of HYZ62 increased Gs by 41.41%, 48.08%, and 17.86%, respectively, compared to NaCl treatment (Figure 5b). The hemin + 0.3%S- and hemin + 1.2%S-treated Gs with 158R increased by 23.44% and 8.62% compared to NaCl stress, but the hemin + 0.6%S treatment led to a decrease of 18.31% (Figure 5c). It is worth noting that the Ci of both rape varieties decreased after hemin soaking, except hemin + 0.6%S of HYZ62, which may be related to the decrease in leaf area after hemin treatment (Figure 5d).

### 3.5. Hemin Seed Soaking Can Reduce Malondialdehyde (MDA) and Electrolyte Leakage Induced by NaCl

**Figure 6:**
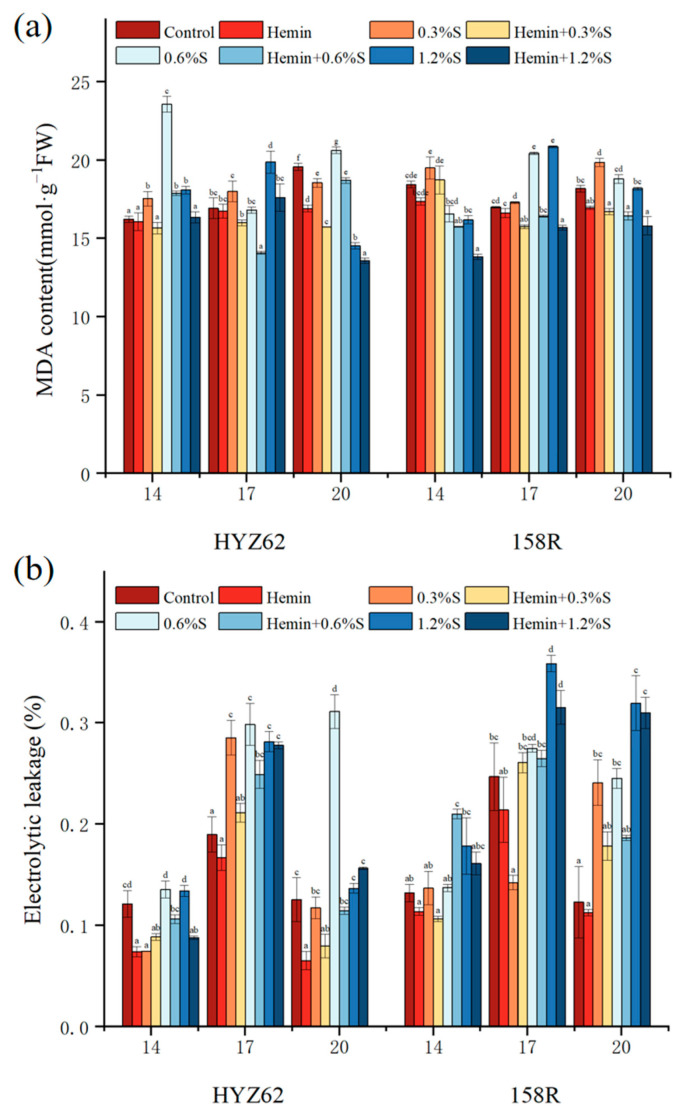
Effects of hemin soaking on MDA (**a**), and EL (**b**) contents of HYZ62 and 158R under NaCl stress (14d). Mean ± SE of three replicates. Different letters indicate significant differences (*p* < 0.05).

The accumulation of malondialdehyde (MDA) indicates that plant cell membranes are damaged and reactive oxygen species are produced under NaCl stress (Figure 6a). Compared to the control, MDA content in HYZ62 was significantly increased under NaCl stress on the 14th day, and the maximum value appeared in 0.6%S treatment. On the 17th day, MDA with 0.3%S treatment and 1.2%S treatment of HYZ62 was significantly increased, and there was no significant change in 0.6%S treatment compared to the control. On day 20, 0.6%S treatment showed significant increases compared to the control, while 0.3%S and 1.2%S treatments showed significant decreases. On day 20, 0.6%S treatment showed a significant increase compared to the control, while 0.3%S and 1.2%S treatments showed a significant decrease.

However, after hemin soaking, MDA content significantly decreased compared to all NaCl treatments (Figure 6a). In NaCl, concentrations of 0.3%, 0.6%, and 1.2%, respectively, were reduced by 10.66–15.22%, 9.34–24.14%, and 6.58–11.40%. Compared to the control, the MDA content of 158R increased on day 14 in 0.3%S treatment, but did not reach a significant level, and all NaCl stress treatments showed an increase on day 17 and day 20. Compared to the control, there was no significant change in the MDA of plants undergoing hemin treatment. Compared to NaCl treatment, the MDA content of seedlings after hemin soaking significantly decreased, and decreased by 3.93–15.77% with 0.3%S treatment, 4.96–19.66% with 0.6%S treatment and 13.1–24.73% with 1.2%S treatments, respectively (Figure 6a).

In the study results, it was observed that electrolyte leakage (EL) significantly damaged the cell membrane (Figure 6b), and the EL of HYZ62 (9.03–48.29%) and 158R (35.08–1.6) increased with NaCl treatment, especially with 1.2%S treatment. Hemin alleviated electrolyte leakage and had a positively effect on the cell structure of the cell membrane (Figure 6b).

### 3.6. Effects of Hemin Seed Soaking on Antioxidant Properties of Rape Leaves under NaCl Stress

**Figure 7:**
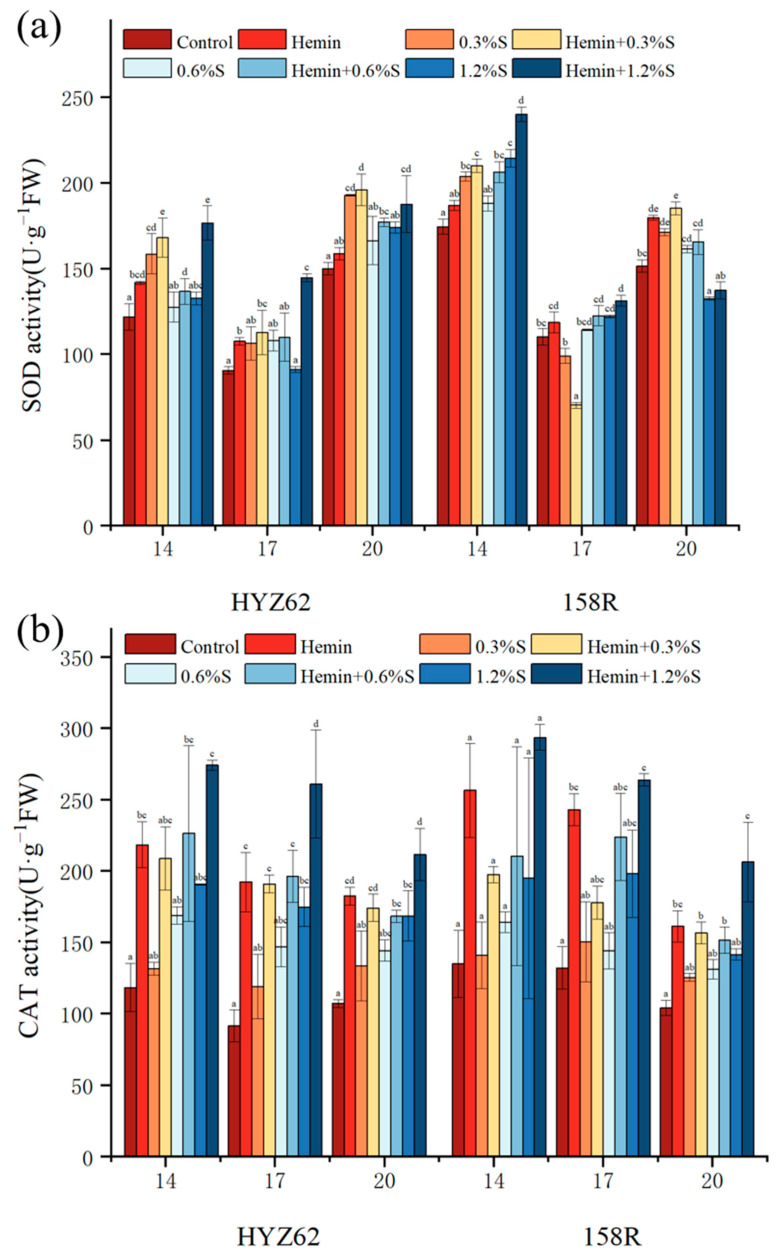

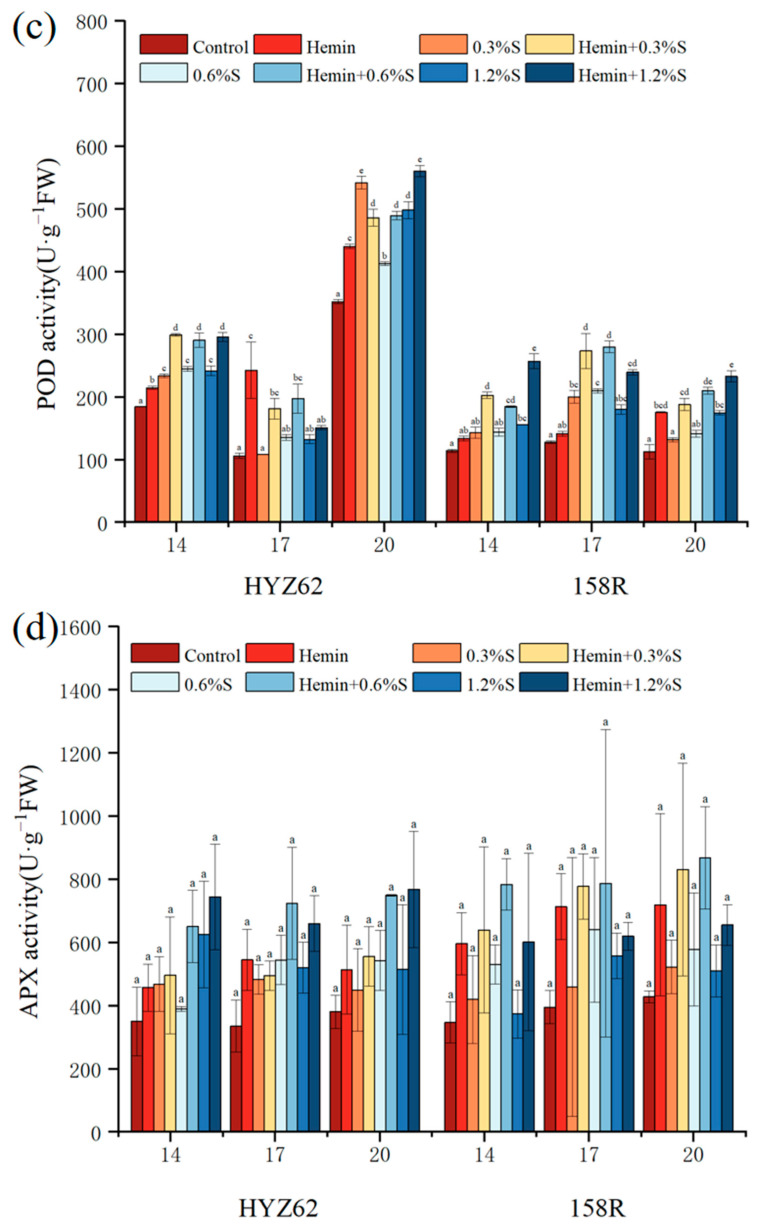
Effects of hemin soaking on the SOD (**a**), POD (**b**), CAT (**c**) and APX (**d**) contents of HYZ62 and 158R under NaCl stress (14d). Mean ± SE of three replicates. Different letters indicate significant differences (*p* < 0.05).

Under salt stress, plants respond to oxidative damage by increasing the synthesis of antioxidant enzymes to protect cells from oxidative damage (Figure 7). In this study, SOD activity significantly increased under NaCl stress. In HYZ62, SOD activity in other NaCl treatments was higher than that in the control treatment, except that there was no significant change in the 1.2%S treatment on day 17. For the SOD activity of 158R, all NaCl treatments were higher than the control on day 14. The 0.3%S treatment on day 17 showed a decrease compared to the control, while the 0.6%S treatment and 1.2%S treatments showed an increase. On day 20, only 1.2%S treatment processing showed a significant decrease. Compared to NaCl treatment, the SOD activity of hemin-treated seedlings was further increased (Figure 7a). SOD activity in the hemin + 1.2%S treatment of HYZ62 significantly increased compared to 1.2%S treatment, and increased by 33%, 58.8%, and 7.8% on the 14th, 17th, and 20th days, respectively. In 158R, hemin + 0.3%S treatment was lower than NaCl treatment on the 17th day. Other hemin treatments increased the SOD activity of rape seedlings, and hemin + 1.2%S treatment had the highest SOD activity on the 14th and 17th days in response to high NaCl stress (Figure 7a). In terms of CAT activity, both rape seedlings of the two varieties were improved under different concentrations of NaCl stress. CAT activity was proportional to NaCl concentration. After hemin soaking, the CAT activity of the seedlings was significantly higher than that following NaCl treatment, and the two cultivars reached the maximum value with hemin + 1.2%S treatment (Figure 7b). Compared to the control, the POD activity of HYZ62 was significantly increased on the 14th and 20th days under NaCl stress, and slightly increased on the 17th day. In 158R, POD activity significantly increased under NaCl stress, with the maximum value occurring with 0.6%S treatment or 1.2%S treatment. Hemin soaking increased POD activity in rapeseed leaves. Compared to NaCl treatment, the POD activity of HYZ62 and 158R significantly increased after hemin soaking (Figure 7c). More importantly, under NaCl stress, APX activity in leaves of two rapeseed varieties significantly increased. After hemin treatment, the APX activity of HYZ62 and 158R leaves was further increased. HYZ62 increased by 2.68–23.55% with hemin + 0.3%S treatment, 6.69–66.87% with hemin + 0.6%S treatment, and 18.97–49.29% with hemin + 1.2%S treatment, and 158R increased by 52.32–69.21% with hemin + 0.3%S treatment, 23–50.13% with hemin + 0.6%S treatment, and 11.14–60.93% with hemin + 1.2%S treatment (Figure 7d).

### 3.7. Effects of Hemin Seed Soaking on Soluble Protein Content in Rape Leaves under NaCl Stress

**Figure 8:**
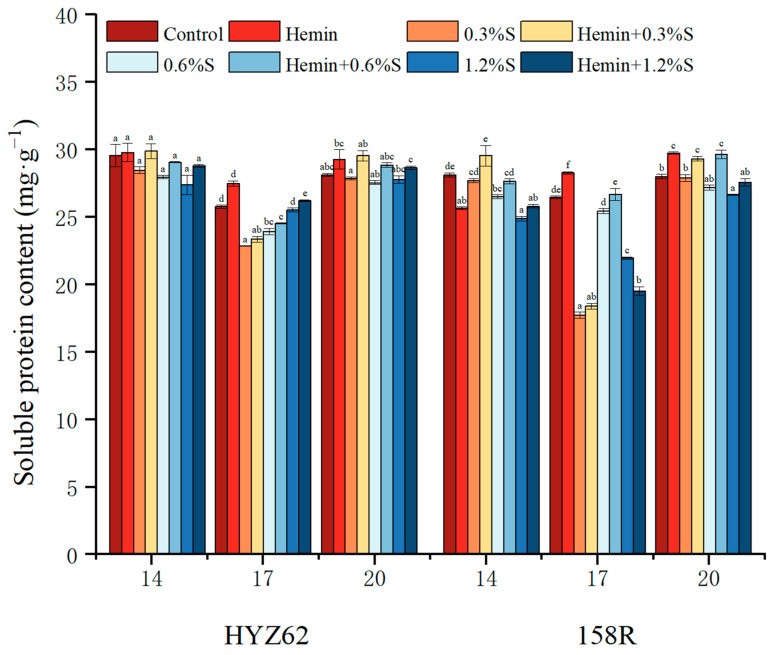
Effects of hemin soaking on the soluble protein content of HYZ62 and 158R under NaCl stress. Mean ± SE of three replicates. Different letters indicate significant differences (*p* < 0.05).

Compared to the control, NaCl stress decreased the soluble protein content in rapeseed leaves (Figure 8). During the sampling period, the soluble protein of the two rapeseed varieties gradually decreased and then increased. On days 14 and 20, HYZ62 and 158R both showed minimum values with the 1.2%S treatment. Compared to NaCl stress, the soluble protein content increased after hemin soaking. The results showed that hemin alleviated NaCl stress by regulating soluble protein content.

## 4. Discussion

**Figure 9:**
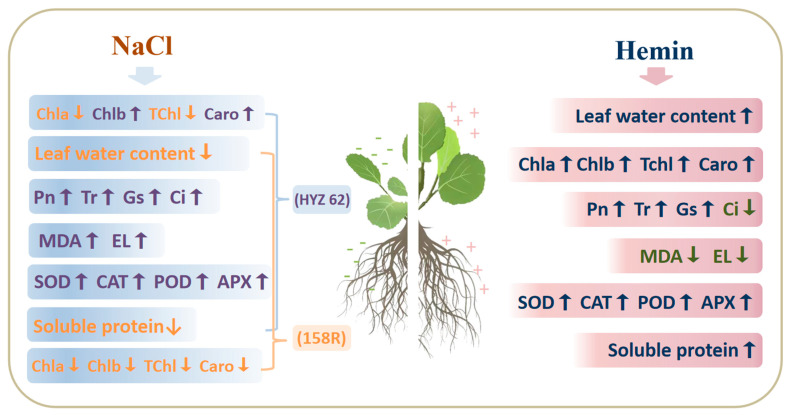
Effects of hemin soaking on HYZ62 and 158R under NaCl stress. “↑” indicate increase or rise, “↓” indicate decrease or decline.

In this study, due to the adverse effects of salt stress, seedlings grew slowly, plant height decreased, and leaf area decreased. To explore the response of rapeseed seedlings to NaCl stress, the exogenous chemical regulation of plants by growth regulators can effectively avoid salt damage. Hemin has been shown to improve plant stress tolerance in different plant species under various abiotic stress conditions [31]. Hemin has been reported to act as an inducer of heme oxygenase-1 (HO-1) at the transcriptional, translational, and post-transcriptional levels upon entry into plants [32], and subsequently decomposes hemin into BV (BR), CO, and Fe^2+^ [33]. In addition, Su et al. proposed that the Cd uptake of reduced heme may depend on its degradation byproducts: Fe^2+^ and CO [34]. As an endogenous signaling molecule, CO plays a role in HO-mediated cellular protection and coordinates cellular defense [35]. Chen et al. [36] showed that iron had an antagonistic effect on the absorption of Zn^2+^ by plants. Therefore, the iron produced by the degradation of heme chloride can potentially reduce the Na^+^ absorption by by plants, thus promoting plant growth (Figure 8 and Figure 9).

### 4.1. Hemin Soaking Promotes Early Growth of Plant Seedlings

Salt stress affected seed germination and inhibited the growth and development of rape seedlings. In this study, NaCl stress resulted in a decrease in seedling height, leaf shrinkage, and biomass accumulation, reflecting the oxidative damage in plants. However, hemin soaking alleviated the damage caused by NaCl stress, which meant that plant height increased, root length elongated, and dry weight increased. This is consistent with the results of the [37] study showing that hemin can prevent a decrease in plant height and dry matter accumulation in wheat seedlings under salt stress. Contrary to the study in [38], he showed that applying hemin improved the succulence and leaf area of barley under salt stress. In this experiment, hemin reduced the leaf area 6666of seedlings, possibly because plants reduced water evaporation and improved water use efficiency by shrinking leaves to adapt to the water loss caused by salt stress.

After salt stress, the underground part of the plant is the first part exposed to salt poisoning, so that this will respond to the stress earlier. Reference [39] pointed out that the root system has flexibility and plasticity and can adjust its shape in time to reduce the absorption of toxic ions. After hemin treatment, the root–shoot ratio of seedlings increased significantly (Table S3), possibly because hemin promoted the growth and development of plants by promoting root growth to absorb more water and nutrients and ensure the normal growth of plants under stress [40]. In addition, the increase in root-to-shoot ratio can also increase the carbon fixation capacity of plants and promote photosynthesis and biomass accumulation [41]. How hemin regulates root growth under NaCl stress requires further study. Salt stress leads to osmotic stress and an imbalance in cell osmotic regulation, thus inhibiting the water absorption capacity of plant roots, resulting in the biocontrolled water absorption of cells, a reduction in the relative water content of leaves, delayed growth, and reduced biomass accumulation [42]. Hemin soaking increases leaf water content, which may be due to the avoidance of osmotic stress caused by salinity conditions, or the high percentage of evaporation caused by the preservation of open stomata [43].

### 4.2. Effects of Exogenous Hemin on the Photosynthetic Parameters of Rape Seedlings under NaCl Stress

The results showed that hemin soaking inhibited the degradation of photosynthetic pigments (Chl a, Chl b and Car) induced by NaCl stress. Zoufan demonstrated that chlorophyll loss or degradation may occur prematurely if triggered by external factors, leading to leaf senescence [44]. In 158R, total chlorophyll and carotenoid contents decreased with the 1.2%S treatment. However, the total chlorophyll and carotenoid contents of HYZ62 increased with 1.2%S treatment, which may be due to the antioxidant properties of chlorophyll, which can help to neutralize and remove active oxidizing substances, thus protecting plant cells from damage [45]. Therefore, plants may increase chlorophyll synthesis under salt stress to enhance antioxidant protection mechanisms. The results showed that HYZ62 promoted the seedlings’ normal growth and development through various mechanisms, and its salt tolerance was better than 158R. The results showed that hemin treatment increased the photosynthetic pigment content in leaves. This may be because hemin regulates the activity of enzymes related to chlorophyll biosynthesis and promotes the protective effect of heme oxygenase 1 (HO-1) on the chromophore of photochromes [46].

The maintenance of photosynthesis is an essential mechanism for plants’ adaptation to salt tolerance [47]. Under NaCl stress, Pn, Gs, Tr, and Ci of HYZ62 showed an increasing trend. Under salt stress, plants can regulate enzyme activity through an antioxidant system, thus increasing the efficiency of photosynthesis. Salt stress may increase the activity of Rubisco, a key enzyme in photosynthesis, thereby increasing the rate of carbon fixation [48]. Pn, Gs, Tr and Ci of 158R showed a decreasing trend. This is due to the physiological drought of plants caused by salt stress, and plants try to reduce their Gs to minimize water loss, resulting in reduced net CO_2_ assimilation, which cannot meet the needs of normal photosynthesis [49]. In this study, the Pn, Gs and Tr of the two rape varieties were significantly increased after hemin soaking, indicating that the reason why hemin effectively alleviates the restriction of leaf stomata induced by NaCl stress may be the maintenance of high levels of Gs and Tr. The increase in Gs is conducive to the exchange of CO_2_ gas into plants and carbon assimilation. The improvement in Tr enhances plants’ transportation ability and their ability to absorb water, which is conducive to photosynthesis and consistent with previous research results [22]. In addition, increasing photosynthetic capacity can promote the synthesis of more organic substances in the leaves. This was confirmed by the increase in dry matter after hemin treatment. The decrease in Ci content after hemin soaking may be due to the decrease in water transpiration and gas exchange by reducing the leaf area, thus reducing the loss of water and energy. This helps plants survive and grow in harsh environments.

### 4.3. Hemin Soaking Induces a Cellular Defense System against Salt Stress

In addition to inhibiting photosynthetic capacity, the REDOX imbalance is another toxic effect of salt on plants. The results showed that NaCl stress increased the MDA content and EL of leaves. Under NaCl stress, plants produce excessive reactive oxygen species, causing the accumulation of membrane lipid peroxides such as MDA, damaging the integrity of cell membranes, and ultimately inhibiting plant growth [50]. In addition, the excessive accumulation of H_2_O_2_ also leads to higher lipid peroxidation, which leads to leakage of cellular components [51]. Plants can inhibit and clear ROS by regulating antioxidant enzyme activity. Therefore, SOD, POD, CAT, and APX contents increased under NaCl stress. It is worth noting that the CAT content of different rapeseed varieties was different under salt stress, and the maximum value of HYZ62 was found with 1.2%S treatment, while that of 158R was found with 0.3%S treatment or 0.6%S treatment [52]. This indicates that HYZ62 has stronger stress resistance under salt stress.

The content of MDA in plant leaves decreased after hemin soaking. Some studies have reported that hemin reduces the relative conductivity and MDA contents, and increases the activity of related antioxidant enzymes [53]. The results showed that the activities of CAT, POD, and APX were increased compared with the control group, while the activity of SOD was not significantly changed. Similar protective effects of heme on oxidative stress have also been observed in soybeans and alfalfa subjected to Al, Cd or UV-B toxicity [54]. Cui [55] showed that hemin reduced the content of thiobarbituric acid (TBARS), regulated the activities of APX, SOD and POD, and alleviated the oxidative damage caused by Cd. In addition, given that iron is present in the structure of CAT and APX, the increase in the activity of CAT and APX after hemin soaking may be related to the release of Fe^2+^ from heme in plants.

### 4.4. Effects of Hemin on Soluble Protein in Rapeseed under NaCl Stress

In osmotic stress, plants can resist injury by increasing macromolecular osmotic substances. As an essential osmotic regulator, soluble protein has strong hydrophilicity and can enhance the water-holding capacity of plant cells [56]. Studies have shown that NaCl stress reduces the soluble protein content in the leaves of two rape varieties. In a high-salt environment, salt ions can form ionic bonds with amino acids in proteins, changing the structure and function of proteins [57]. As a result, protein synthesis is biocontrolled, and soluble protein synthesis is reduced. Second, under salt stress, plants may respond to stress by accelerating the degradation of existing proteins to release amino acids for other physiological processes [58]. This leads to the increased degradation of soluble proteins, reducing soluble protein content. Hemin soaking can effectively improve the soluble protein content of the two rape varieties and help the cells absorb water in the external environment, thus reducing the damage caused by NaCl stress. This confirms that hemin enhances the osmoprotector [59], thereby inhibiting oxidative-damage-induced growth by removing excess ROS and REDOX homeostasis from the electron transport chain.

## 5. Conclusions

In conclusion, NaCl stress, especially 1.2%S, seriously affected the normal growth and development of HYZ62 and 158R rapeseed seedlings. Hemin soaking significantly effected the morphology and physiology of rapeseed seedlings under NaCl stress. Hemin soaking can reduce Na^+^ toxicity under NaCl stress and promote the germination and early seedling growth of two rapeseed varieties, increased the root–crown ratio of the plants, and promoted water uptake. In addition, hemin soaking can promote the growth of rapeseed by enhancing photosynthesis, improving antioxidant enzyme activity, and the osmotic regulation ability of seedlings to resist salt stress. The results of this study demonstrated that the use of hemin chloride seed dipping can improve abiotic stress tolerance in oilseed rape to cope with saline environments. In addition, hemin can be used as an effective inducer to solve the technical difficulties of traditional agronomic measurements that are difficult to implement. How hemin reduces salinity toxicity in plants requires further research.

## Figures and Tables

**Table 1:** The effect of hemin on the morphological growth of rapeseed seedlings under NaCl stress.

Index	Treatments	HYZ 62			158R		
		14	17	20	14	17	20
Shoot Length (cm)	Control	15.23 ± 0.22 e	15.37 ± 0.03 c	21.67 ± 0.33 f	18.57 ± 0.23 e	20.20 ± 0.36 d	20.80 ± 0.81 bc
	Hemin	14.93 ± 0.07 e	16.30 ± 0.21 d	18.83 ± 0.32 cd	16.93 ± 0.47 d	20.10 ± 0.26 d	23.23 ± 0.43 d
	0.3%S	12.87 ± 0.30 c	16.33 ± 0.17 d	20.33 ± 0.33 e	16.17 ± 0.17 d	17.77 ± 0.37 c	22.60 ± 0.31 cd
	Hemin + 0.3%S	14.33 ± 0.09 c	17.00 ± 0.00 e	18.17 ± 0.09 c	14.93 ± 0.07 c	15.27 ± 0.38 b	21.33 ± 0.67 bcd
	0.6%S	10.73 ± 0.12 b	12.63 ± 0.35 a	19.77 ± 0.23 de	12.90 ± 0.06 b	14.63 ± 0.18 ab	19.63 ± 0.12 b
	Hemin + 0.6%S	12.47 ± 0.03 c	14.17 ± 0.09 b	21.33 ± 0.67 f	13.70 ± 0.15 b	17.43 ± 0.22 c	21.07 ± 0.13 bcd
	1.2%S	9.83 ± 0.20 a	13.00 ± 0.40 d	13.10 ± 0.10 a	10.33 ± 0.20 a	13.70 ± 0.30 a	16.90 ± 0.31 a
	Hemin + 1.2%S	10.60 ± 0.00 b	14.57 ± 0.15 e	15.00 ± 0.00 b	12.67 ± 0.17 b	15.47 ± 0.23 b	20.40 ± 0.30 b
	Control	16.97 ± 0.33 d	19.50 ± 0.26 c	18.5 ± 0.29 de	17.33 ± 0.17 b	17.83 ± 0.41 abc	18.63 ± 0.43 abc
	Hemin	17.50 ± 0.00 d	18.83 ± 0.18 c	19.40 ± 0.44 e	19.80 ± 0.30 d	20.03 ± 0.23 c	17.90 ± 0.38 ab
Root length (cm)	0.3%S	15.47 ± 0.47 bc	16.47 ± 0.34 a	16.77 ± 0.39 abc	16.53 ± 0.23 b	17.77 ± 0.15 abc	17.97 ± 0.26 ab
	Hemin + 0.3%S	15.87 ± 0.13 bc	17.30 ± 0.31 b	18.47 ± 0.64 de	19.93 ± 0.30 d	18.70 ± 1.05 bc	20.93 ± 0.56 c
	0.6%S	15.10 ± 0.06 b	16.03 ± 0.23 a	16.27 ± 0.35 ab	16.83 ± 0.17 b	16.53 ± 0.23 ab	16.90 ± 0.47 a
	Hemin + 0.6%S	16.50 ± 0.29 cd	17.33 ± 0.19 b	17.43 ± 0.30 bcd	18.93 ± 0.58 cd	19.40 ± 0.00 c	19.93 ± 0.68 bc
	1.2%S	13.47 ± 0.55 a	15.93 ± 0.23 a	15.73 ± 0.43 a	14.83 ± 0.17 a	16.03 ± 0.32 a	16.53 ± 0.20 a
	Hemin + 1.2%S	15.80 ± 0.42 bc	17.80 ± 0.06 b	17.83 ± 0.67 cd	17.83 ± 0.33 bc	18.50 ± 0.55 bc	21.23 ± 1.01 c
	Control	3497.1 ± 146.0 e	3922.9 ± 70.5 e	5381.5 ± 59.6 ef	2634.9 ± 122.6 d	4636.2 ± 69.5 c	5086.3 ± 354.2 bc
	Hemin	2772.7 ± 85.1 d	3388.1 ± 28.9 d	4948.8 ± 265.2 de	2462.4 ± 228.5 cd	4179.3 ± 114.8 bc	6514.1 ± 61.5 d
Leaf area (cm^2^)	0.3%S	2781.2 ± 111.7 d	2836.2 ± 81.7 bc	5823.9 ± 259.1 f	2581.0 ± 50.2 d	3782.0 ± 66.2 b	5973.4 ± 26.4 cd
	Hemin + 0.3%S	2449.4 ± 48.7 c	3463.3 ± 136.5 d	4167.6 ± 71.5 bc	2277.8 ± 266.4 bcd	2832.1 ± 75.1 a	5309.4 ± 329.6 bc
	0.6%S	1677.5 ± 16.2 b	3094.8 ± 179.8 cd	4577.8 ± 101.9 cd	1715.8 ± 52.3 abc	2839.1 ± 29.1 a	4301.9 ± 102.1 ab
	Hemin + 0.6%S	1756.9 ± 37.6 b	2670.1 ± 65.6 ab	3986.9 ± 214.1 b	1889.0 ± 78.1 abcd	3630.5 ± 39.7 b	4881.3 ± 312.9 bc
	1.2%S	1324.7 ± 12.7 a	2678.8 ± 203.1 ab	2786.7 ± 125.3 a	1222.2 ± 161.3 a	2698.5 ± 268.7 a	3518.8 ± 120.1 a
	Hemin + 1.2%S	1130.7 ± 6.3 a	2338.4 ± 38.1 a	3281.6 ± 86.8 a	1616.2 ± 122.0 ab	2930.6 ± 173.0 a	5230.5 ± 219.8 bc

Mean ± SE of three replicates. Different letters indicate significant differences (*p* < 0.05).

## Data Availability

Data may be obtained through the corresponding author for reasonable reasons.

## Supplementary Materials

The following supporting information can be downloaded at: https://www.mdpi.com/article/10.3390/metabo14010057/s1, Table S1: Effect of hemin soaking on the seedling emergence rate of HYZ62 and 158R under NaCl stress; Table S2: Effect of hemin soaking on fresh weight of HYZ62 and 158R under NaCl stress; Table S3: Effect of hemin soaking on the root–shoot ratio of HYZ62 and 158R under NaCl stress.
