# Supplementary material for: Metabolic Adaptations in Rapeseed: Hemin-Induced Resilience to NaCl Stress by Enhancing Growth, Photosynthesis, and Cellular Defense Ability
Source: Metabolites. 2024 Jan 15;14(1):57. doi: 10.3390/metabo14010057 (PMC10818378; doi:10.3390/metabo14010057)
Supplement: Supplementary file 1 [file metabolites-14-00057-s001.zip › metabolites-2778695-supplementary.pdf]

Table S1: Effect of hemin soaking on the seedling emergence rate (%) of HYZ62 and 158R under NaCl stress

Treatments	Huayouza 62	158r
Control	0.818	0.705
Hemin	0.890	0.870
0.3%S	0.655	0.691
Hemin+0.3%S	0.825	0.700
0.6%S	0.850	0.736
Hemin+0.6%S	0.915	0.900
1.2%S	0.540	0.390
Hemin+1.2%S	0.710	0.565

Table S2:Effect of hemin soaking on fresh weight of HYZ62 and 158R under NaCl stress

Index	Treatments	Huayouza 62			158R		
		14	17	20	14	17	20
Shoot fresh weight (g)	Control	1.3240±0.0782e	1.7757±0.0553ef	2.3227±0.0863c	1.2217±0.0919cd	1.8760±0.0354d	2.6750±0.0922cd
	Hemin	1.1387±0.0251d	1.8523±0.0263f	1.8950±0.0824b	1.4090±0.0625d	2.0623±0.0048d	2.9303±0.0468de
	0.3%S	0.9120±0.0565c	1.5480±0.0600d	2.7043±0.1077d	1.2350±0.0185cd	2.0073±0.0552d	2.5813±0.1875cd
	Hemin+0.3%S	1.0447±0.0358d	1.6803±0.0596de	2.3417±0.1181c	1.1553±0.0120c	1.5520±0.0620c	3.2713±0.1118e
	0.6%S	0.7467±0.0054b	1.1483±0.0171a	2.0803±0.1696bc	0.7917±0.0297b	1.1240±0.0100a	2.0527±0.0238b
	Hemin+0.6%S	0.7510±0.0182b	1.3300±0.0083c	2.1100±0.0778bc	0.7783±0.0113b	1.5150±0.0752c	2.3423±0.0231bc
	1.2%S	0.4693±0.0073a	1.1797±0.0691ab	1.1413±0.0326a	0.5093±0.0057a	1.2010±0.0050ab	1.3400±0.0576a
	Hemin+1.2%S	0.5490±0.0197a	1.2943±0.0198bc	1.3543±0.0177a	0.7660±0.0402b	1.3963±0.0317bc	2.4067±0.0925bc
Root fresh weight (g)	Control	0.1317±0.0015d	0.1540±0.0045d	0.3200±0.0090de	0.1340±0.0066bc	0.1513±0.0018a	0.2843±0.0048c
	Hemin	0.1820±0.0057e	0.1760±0.0122e	0.3517±0.0106e	0.2097±0.0168d	0.1850±0.0067bc	0.3687±0.0124de
	0.3%S	0.1017±0.0035c	0.1283±0.0091c	0.2720±0.0520cd	0.1263±0.0073bc	0.1593±0.0116ab	0.2880±0.0191cd
	Hemin+0.3%S	0.1067±0.0080c	0.1530±0.0067c	0.3800±0.0060e	0.1440±0.0046c	0.2070±0.0065c	0.3830±0.0067e
	0.6%S	0.0770±0.0066b	0.1093±0.0086b	0.2280±0.0115bc	0.1010±0.0012ab	0.1340±0.0071a	0.1713±0.0234ab
	Hemin+0.6%S	0.1033±0.0077c	0.1163±0.0012c	0.2740±0.0300cd	0.1453±0.0055c	0.2083±0.0043c	0.2410±0.0096bc
	1.2%S	0.0533±0.0052a	0.0950±0.0040a	0.1247±0.0264a	0.0777±0.0062a	0.1507±0.0073a	0.1287±0.0252a
	Hemin+1.2%S	0.0713±0.0041b	0.1380±0.0055b	0.1750±0.0023ab	0.1270±0.0020bc	0.1997±0.0052c	0.2827±0.0181c

Mean ± SE of three replicates. Different letters indicate significant differences ($p < 0.05$)

Table S3: Effect of hemin soaking on root-shoot ratio of HYZ62 and 158R under NaCl stress

Index	Treatments	Huayouza 62			158R		
		14	17	20	14	17	20
root-shoot ratio (%)	Control	0.1247±0.0052a	0.1357±0.0020ab	0.1233±0.0038ab	0.1047±0.0055a	0.1230±0.0015ab	0.1143±0.0018abc
	Hemin	0.1730±0.0017c	0.1503±0.0020c	0.1460±0.0055c	0.1413±0.0009cd	0.1433±0.0003cd	0.1367±0.0080d
	0.3%S	0.1217±0.0038a	0.1360±0.0021ab	0.1327±0.0072bc	0.1020±0.0017a	0.1157±0.0032a	0.1023±0.0009a
	Hemin+0.3%S	0.1580±0.0032b	0.1390±0.0006b	0.1530±0.0050c	0.1280±0.0017bc	0.1533±0.0059de	0.1257±0.0019cd
	0.6%S	0.1517±0.0029b	0.1263±0.0013a	0.1020±0.0032a	0.1163±0.0027ab	0.1423±0.0007cd	0.1147±0.0018abc
	Hemin+0.6%S	0.1883±0.0027d	0.1383±0.0026b	0.1167±0.0052ab	0.1370±0.0015cd	0.1613±0.0062e	0.1290±0.0006cd
	1.2%S	0.1753±0.0052c	0.1683±0.0013d	0.1120±0.0136ab	0.1513±0.0058d	0.1340±0.0031bc	0.1073±0.0012ab
	Hemin+1.2%S	0.1940±0.0031d	0.1723±0.0079d	0.1323±0.0030bc	0.1733±0.0041e	0.1613±0.0032e	0.1217±0.0012bcd

Mean ± SE of three replicates. Different letters indicate significant differences ($p < 0.05$)
